# Prevalence and Temporal Dynamics of White Line Disease in Sheep: An Exploratory Investigation into Disease Distribution and Associated Risk Factors

**DOI:** 10.3390/vetsci8060116

**Published:** 2021-06-19

**Authors:** Caroline M. Best, Janet Roden, Kate Phillips, Alison Z. Pyatt, Malgorzata C. Behnke

**Affiliations:** 1 Department of Veterinary Health & Animal Sciences, Harper Adams University, Newport, Shropshire TF10 8NB, UK; jroden@harper-adams.ac.uk (J.R.); kphillips@harper-adams.ac.uk (K.P.); mbehnke@harper-adams.ac.uk (M.C.B.); 2 International Office, Veterinary Medicines Directorate, Addlestone, Surrey KT15 3NB, UK; a.pyatt@vmd.gov.uk

**Keywords:** lameness, sheep, white line disease, shelly hoof, white line, prevalence, disease dynamics, risk factors

## Abstract

Lameness in sheep is a global health, welfare and economic concern. White line disease (WLD), also known as shelly hoof, is a prevalent, non-infectious cause of lameness, characterised by the breakdown of the white line. Little is known about the predisposing factors, nor the individual disease dynamics over time. Our exploratory study aimed to investigate the prevalence and temporal dynamics of WLD, and the associated risk factors. Feet of 400 ewes from four UK commercial sheep farms were inspected for WLD at four time points across 12 months. The change in WLD state at foot-level (develop or recover) was calculated for three transition periods. We present WLD to be widespread, affecting 46.8% of foot-level and 76.6% of sheep-level observations. States in WLD changed over time, with feet readily developing and recovering from WLD within the study period. The presence of WLD at foot-level, the number of feet affected at sheep-level and dynamics in development and recovery were driven by a variety of foot-, sheep- and farm-level factors. We provide key insight into the multifaceted aetiology of WLD and corroborate previous studies demonstrating its multifactorial nature. Our study highlights an opportunity to reduce WLD prevalence and informs hypotheses for future prospective studies.

## 1. Introduction

Lameness in sheep is a major health and welfare issue affecting >90% of UK flocks [1,2]. Lame sheep are in pain [3] and have compromised productivity [4]. Accordingly, lameness is estimated to cost the UK sheep industry between £24 to £80 million per year in related costs and production losses [4,5]. Lameness in sheep can be attributed to infectious or non-infectious origins. The major infectious lameness type, footrot (FR), is caused by the bacterium *Dichelobacter nodosus*, and has two clinical presentations, interdigital dermatitis (ID) and severe footrot (SFR), which accounts for approximately 70% of lameness and affects around 95% of English flocks [6]. Contagious ovine digital dermatitis (CODD) is another infectious foot disease of concern, caused by *Treponema* bacteria, and affects approximately 50% of UK flocks [7,8].

**Figure 1:**
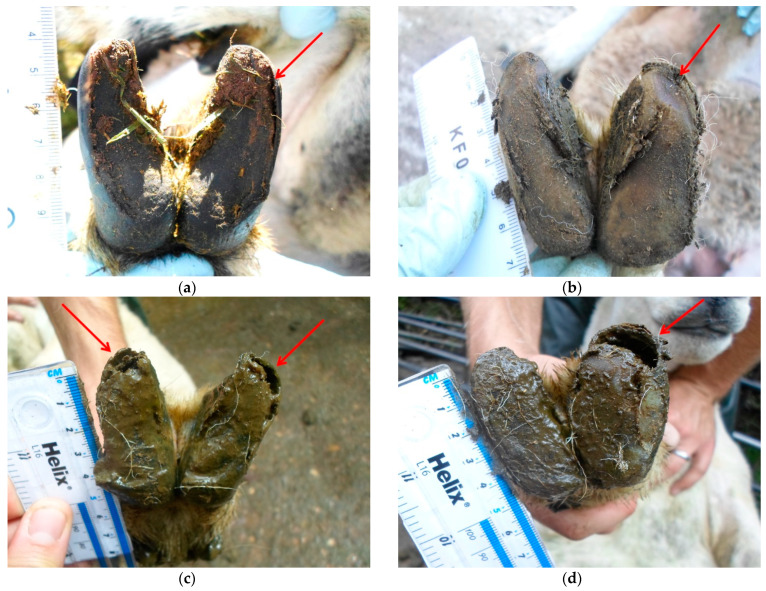
(**a**) Discrete separation of the abaxial hoof wall at the white line (red arrow); (**b**) Discrete discolouration and separation of the abaxial hoof wall at the white line (red arrow); (**c**) Extensive separation at the toe and along the abaxial hoof wall on both paired claws (red arrows); (**d**) Major cavity ‘pocket’ with loss of hoof wall integrity (red arrow).

Additional to infectious aetiologies, lameness types of non-infectious origin are also significant. White line disease (WLD), also known as shelly hoof, accounts for approximately 20% of all hoof lesions in sheep [6]. WLD occurs due to a breakdown of the white line, or the junction between the hard wall horn and the more pliable sole horn of the hoof capsule [9], causing discolouration, fragility and a progressive separation of the hoof wall from the underlying laminae [10,11]. The white line area has a lower puncture resistance than the sole horn, reducing its structural integrity and strength [12]. The wall horn of a WLD-affected foot is characterised by fragmented, poorly keratinised epithelial horn cells with microfissures, with ‘unzipping’ of cell membranes causing deep crevices in the horn [10]. The presentations of WLD typically range from small discrete lesions to major cavity ‘pockets’ causing loss of hoof wall [11] (Figure 1). WLD can be highly prevalent within flocks, in some cases affecting up to 91% of ewes [13] and is anecdotally described as a significant concern to farmers. However, it is currently unknown whether feet become persistently affected once they have developed WLD, or whether patterns of WLD development, recovery and recurrence occur over time. Further work is required to follow the development of WLD longitudinally to provide a novel angle on previous research.

**Figure 2:**
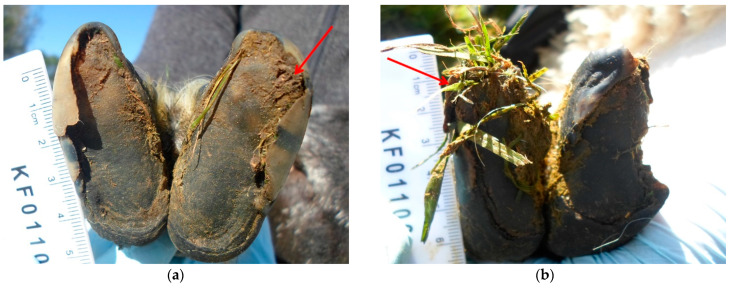
(**a**) Large cavity along the abaxial hoof wall impacted with soil (red arrow); (**b**) Large cavity at the toe and along the abaxial hoof wall impacted with grass and faecal material (red arrow).

In cattle, WLD is analogous to that in sheep and is one of the most frequently recorded hoof diseases affecting modern dairy herds [14,15]. In sheep, WLD only amounts to <3% of lameness in flocks [6,16,17], but hooves affected by WLD can become severely damaged, causing considerable pain and discomfort [13]. The resultant weakened white line is susceptible to foreign material being caught in the distal surface [9], and acute lameness arises when cavities become impacted with foreign material (Figure 2), such as soil and stones, and opportunistic bacteria, leading to secondary infection and inflammation. Infections can progress to toe abscessation with pus forming in the laminae underneath the hoof wall [11]. Bacterial invasion is also facilitated by deep crevices in the hoof wall caused by WLD [11]. However, no studies have reported an association between infectious lameness types and the presence of WLD. Nonetheless, the presence of WLD lesions is important as a potential cause of lameness and has implications on lameness control in flocks.

A range of predisposing factors are implicated in WLD development, although the exact causes of WLD are presently unknown. From retrospective farmer-reported estimates of WLD, footbathing (with formalin) and foot trimming were identified as key risk factors for WLD [18]. These farm-level managements, increasing the risk of damage to the white line, could explain differences in prevalence observed between farms [13]. Sheep grazing poor quality pasture has also been reported to increase the risk of WLD [18], highlighting the possible role of nutritional deficiency in WLD occurrence [10]. Furthermore, previous work has alluded to seasonal differences in WLD prevalence on farms, with peak prevalence occurring during the summer months [13]. Susceptibility or resistance to WLD has been identified at sheep-level; whether sheep have WLD is under moderate genetic control [10], with some genotypes and individual alleles being linked to susceptibility or resistance to WLD [13]. Furthermore, significant between-breed variation in WLD prevalence has been reported [19], indicating that variation in hoof structural properties between breeds can impact the expression of WLD. In cattle, separation of the white line is considered to be the combined result of weakened hoof suspensory apparatus from laminitis, poor horn quality, anatomical predisposition and environmental factors [20]. Laminitis in sheep is thought to be a prevalent, yet under or misdiagnosed condition [21], but is an important risk factor for WLD in cattle [22]. Whilst we acknowledge that the aetiology of WLD is multifactorial and complex, there is a lack of understanding of the factors implicated in both the development and recovery from WLD. Further work is required to investigate the sheep- and farm-level factors associated with foot susceptibility to WLD, to better understand the risk factors affecting individual disease dynamics.

The aims of our study were to describe in detail the prevalence of WLD at foot- and sheep-level in commercial ewes, and to provide novel insight into the risk factors for WLD. Furthermore, we sought to explore the dynamics in WLD disease states across three transition periods and the risk factors associated with the development of or recovery from WLD. To our knowledge, this is the first repeated cross-sectional field survey investigating the prevalence and temporal dynamics of WLD in commercial sheep flocks.

## 2. Materials and Methods

### 2.1. Study Design

The study was a longitudinal, repeated cross-sectional field survey of four commercial sheep farms (identified as A–D) in England and Wales (Table 1). Additional farm characteristics, pertinent to the study, are presented in the results section. Farms were convenience selected based on their location, and farmers’ willingness to participate in the study. Only farms where ewes’ feet were not routinely trimmed, nor routinely footbathed, were selected for inclusion.

At the start of the study, a minimum of 90 ewes were initially convenience selected from each flock, ensuring distribution between two age groups: <4 years and ≥4 years. All ewes were individually identified by ear tag numbers and marked for inclusion in the study. Due to the dynamic nature of commercial sheep flocks, not all ewes were present at all visits. Therefore, previously unsampled ewes were included into the study, where possible. All ewes were managed as part of the main flock throughout the study period. Each farm was visited four times across a 12-month period; September 2019 (visit 1), January 2020 (visit 2), July 2020 (visit 3) and September 2020 (visit 4).

### 2.2. Data Collection

All sheep-level data were recorded by a single observer (CMB) on paper recording sheets. Ewe age was recorded at the start of the study. At each visit, the study ewes were visually assessed for lameness using a four-point scoring system [23], and scored for body condition (BCS) using a 1–5 scale with 0.5 increments [24]. Whilst ewes were held in a seated position, all eight individual claws were assessed for the presence of WLD, as per previous detailed descriptions [11]. Other clinical diseases were also recorded if present.

Farm-level data were obtained from retrospective management practice questionnaires completed by each farmer. This included flock size and soil type. Assessments of average pasture quality, moisture, type, and sward height were provided for each calendar month between August 2019 and September 2020, similar to the variables assessed previously [25]. Questions were accompanied by guidance to ensure farmers interpreted questions consistently. Meteorological data for each farm were extracted from publicly available local UK MET Office station archives. This included total rainfall (mm) and average daily maximum temperature (°C) for each calendar month. Location of MET office weather stations are presented in Supplementary Table S1.

Only ewes with observations from ≥2 consecutive visits were retained in the final data set. A total of 415 ewes were sampled throughout the duration of the study. Fifteen ewes (3.6%) were sampled either across <2 visits or non-consecutive visits and were excluded from analyses. The majority (61%, *n* = 244/400) of ewes were aged <4 years. On average, the ewes were aged 3.3 years (95% CI: 3.2–3.5 [range: 1–8]). Throughout the study, ewes were sampled, on average, 3.5 times (95% CI: 3.47–3.62). The majority (*n* = 287/400, 71.8%) of ewes were sampled across all four visits (Table 2).

### 2.3. Data Preparation

All data were manually entered into Microsoft Excel 2016 (Microsoft Corporation, Redmond, WA, USA).

The final dataset comprised of 5672 foot-level observations and 1418 sheep-level observations obtained from 400 ewes across four farms. The presence of WLD at foot-level (front left, front right, back left, back right) was defined as ≥1 paired claw (medial and/or lateral) with WLD present. The presence of WLD at sheep-level was defined as ≥1 foot with WLD present. Due to the low prevalence of SFR, the presence of ID and SFR were combined into one category for analysis. Due to the low prevalence of lameness in ewes sampled, locomotion score was omitted from analyses.

From the study, three transition periods were defined: T1—transition period between visits 1 and 2 (September 2019 to December 2020); T2—transition period between visits 2 and 3 (December 2020 to July 2020); and T3—transition period between visits 3 and 4 (July 2020 to September 2020). Transition period lengths were not equal due to farm access restrictions.

For each transition period, the change in WLD state at foot-level was assigned. Four transition states were defined: ‘remain healthy’, when the foot had no WLD present at both visits; ‘remain with WLD’, when the foot had WLD present at both visits; ‘recover’, when the WLD-affected foot was healthy at the subsequent visit; and ‘develop WLD’, when the healthy foot had WLD present at the subsequent visit.

### 2.4. Statistical Analysis

All analyses were conducted in Genstat (VSN International, Hemel Hempstead, UK), and R statistical software package v 3.5.3 (R Foundation for Statistical Computing, Vienna, Austria). Associations between categorical variables were investigated using Chi-squared tests. Associations between continuous and categorical variables were investigated using Kruskal–Wallis tests. Probability values of <0.05 were considered significant.

#### 2.4.1. Associations with the Presence of White Line Disease at Foot-Level

Univariable and multivariable associations with the presence of WLD at foot-level were investigated using binomial mixed effects models. The models were constructed using the “glmer” function from the “lme4” package in R [26], using the binary outcome variable ‘presence of WLD’, categorised as absent or present. To account for repeated observations over time of feet and feet clustered in ewes, ‘Ewe’ was included as a random effect. ‘Farm’ was also included as a random effect to account for clustering of ewes within farm.

Candidate foot-, sheep- and farm-level variables are presented in Table 3. Where appropriate, farm-level variables were also lagged one month previously and included in analyses. Only ewes from Farm B were grazing at visit 2 (January 2020), therefore farm-level variables (pasture, sward and meteorological data) for all farms for the calendar month of visit 2 were excluded from analyses.

The variables were first sequentially tested in univariable models, before constructing the multivariable model using a backward elimination procedure, until only significant variables remained. Only variables with *p* < 0.2 at univariable level were selected to develop the multivariable model. Models with the lowest Akaike Information Criterion (AIC) were favoured. Collinearity was tested by assessing Variance Inflation Factors (VIF) using the “car” package [27].

#### 2.4.2. Associations with the Number of Feet Affected by White Line Disease at Sheep-level (Ewes Affected by WLD Only)

Univariable and multivariable associations with the number of feet affected by WLD at sheep-level were investigated using linear mixed effects models. The models were constructed using the “lmer” function from the “lme4” package in R, using the continuous outcome variable ‘number of feet affected by WLD’ (coded 1–4). The fixed effects considered in the models are presented in Table 3, omitting foot-level variables, but including ‘clinical disease’ at sheep-level. Random factors were ‘Ewe’ and ‘Farm’. The relative fit of models was assessed using AIC values, as described previously. Residual plots were inspected to identify outliers and to ensure the normality assumption was met.

#### 2.4.3. Associations with the Development of, and Recovery from, White Line Disease at Foot-Level

Univariable and multivariable associations with the transition in states of WLD at foot-level were investigated using binomial mixed effects models. Separate models were constructed using the “glmer” function to investigate associations with the two binary outcome variables, (1) ‘Develop WLD’; categorised as ‘remain healthy’ or ‘develop WLD’ and (2) ‘Recover from WLD’; categorised as ‘remain with WLD’ or ‘recover from WLD’. Models for each binary outcome variable were repeated for each transition period, to achieve a maximum of six final multivariable models. Models were constructed as described previously.

Candidate foot-, sheep- and farm-level variables are presented in Table 4. Where appropriate, variables for both the first calendar month of the transition period and for the last calendar month of the transition period, were included in the analyses. Only ewes from Farm B were grazing at visit 2 (January 2020), therefore farm-level variables (pasture, sward and meteorological variables) for all farms for the first calendar month of T2 and the last calendar month of T1 were excluded from analyses.

## 3. Results

### 3.1. Descriptive Results

#### 3.1.1. Prevalence of White Line Disease at Foot-Level

Distribution of WLD at foot-level by foot position, claw position, farm and visit, are presented in Table 5. WLD was observed in 46.8% (*n* = 2657/5672) of foot-level observations. The frequency of WLD was highest in back feet, compared to front feet (*p* < 0.001), and highest in back lateral claws, but lowest in front lateral claws (*p* < 0.001). The majority of feet affected by WLD (57.8%, *n* = 1536/2657) had WLD present in both claws. Back feet were more likely to have both claws affected by WLD, than front feet (*p* < 0.001). Frequency of WLD was highest in feet of sheep on Farm D, but lowest in feet of sheep on Farm C (*p* < 0.001). The frequency of WLD was highest at visits 2 and 4 (*p* < 0.001).

#### 3.1.2. Number of Feet Affected by White Line Disease at Sheep-Level

Throughout the study, 76.6% (*n* = 1086/1418) of sheep-level observations had ≥1 feet affected by WLD. Of the ewes observed at all four visits, 34.8% (*n* = 100/287) were affected by WLD on ≥1 feet on all four occasions. On average, ewes affected by WLD (*n* = 1086) had 2.4 (95% CI: 2.38–2.51) feet affected. The number of feet affected by WLD per ewe differed by farm and visit (Table 6). Ewes on Farm D had the most feet affected by WLD, whereas ewes on Farm C had the least number of feet affected (*p* < 0.001). Ewes sampled at Visit 4 had the most feet affected by WLD, whereas ewes at Visit 3 had the least number of feet affected (*p* < 0.001). WLD was observed on ≥1 feet, on ≥1 occasion for 396 sheep; four ewes (1%) had no WLD during the study period. Approximately 44% (*n* = 628/1418) of sheep-level observations had WLD in at least one front foot and hind foot concurrently.

#### 3.1.3. Transitions in States of White Line Disease

All ewes in the final dataset were observed across ≥1 transition periods (Table 7). A total of 4036 foot-level observations of WLD were observed from 400 ewes over three transition periods.

The distribution of foot-level WLD transition states for each transition period are presented in Table 8. The most common transition was to develop WLD (30.5%, *n* = 1231/4036). Recovery from WLD was most likely to occur during T2, whereas feet were less likely to develop WLD during this period (*p* < 0.001). Within T1, 12.7% (*n* = 194/1528) of feet recovered from WLD. Of those that remained with WLD, 54.4% (*n* = 174/320) recovered in T2, and of those still affected, 16.2% (*n* = 16/99) recovered during T3. Approximately 4% (*n* = 67/1528) of feet from 55 individual ewes (13.8%) with WLD at T1 were affected throughout all three transition periods.

Back feet were more likely to remain with WLD during T2, whereas front feet were less likely to develop WLD (*p* = 0.006). During T3, front feet were more likely to remain healthy, whereas back feet were more likely to remain with WLD (*p* < 0.001). During T1, feet of ewes on Farm A were less likely to develop WLD, whereas feet on Farm B were more likely to develop WLD (*p* < 0.001). During T2, feet of ewes on Farm C were more likely to remain healthy, whereas feet on Farm D were more likely to remain with WLD (*p* < 0.001). During T3, feet of ewes on Farm C were less likely to remain with WLD, whereas feet on Farm D were more likely to remain with WLD (*p* < 0.001).

### 3.2. Associations with the Presence of White Line Disease at Foot Level

Univariable associations with the presence of WLD at foot-level are presented in Supplementary Table S2. Seven variables remained in the final multivariable model (Table 9). Feet of ewes aged ≥4 years had an increased risk of WLD, compared to those of ewes aged <4 years (OR: 1.39, 95% CI: 1.19–1.62). Back feet had an increased risk of WLD, compared to front feet (OR: 1.44, 95% CI: 1.26–1.64). Furthermore, a foot had an increased risk of WLD if ≥1 other feet of the ewe were affected by WLD. Feet of ewes had a reduced risk of WLD when grazing damp (OR: 0.59, 95% CI: 0.44–0.79), or wet pasture (OR: 0.52, 95% CI: 0.36–0.75), compared to dry pasture. Furthermore, feet of ewes grazing average pasture quality one month previously had a reduced risk of WLD (OR: 0.50, 95% CI: 0.42–0.60), but an increased risk when grazing poor pasture one month previously (OR: 2.53, 95% CI: 1.78–3.60), compared to those grazing lush pasture. Feet of ewes grazing longer pasture (approximately 8 cm) had an increased risk of WLD (OR: 1.55, 95% CI: 1.09–2.22), compared to those grazing shorter swards (approximately 3 cm). Feet of ewes had an increased risk of WLD at Visit 2 (OR: 3.99, 95% CI: 2.44–6.53) and Visit 4 (OR: 1.87, 95% CI: 1.24–2.83), but a reduced risk at Visit 3 (OR: 0.41, 95% CI: 0.27–0.63), compared to Visit 1.

Associations with the presence of WLD at sheep-level were also investigated. Three variables remained in the final multivariable model; ‘visit’, ‘sward height’ for calendar month of visit, and ‘pasture quality’ lagged to previous calendar month. All three variables had similar odds ratios and significance as for the presence of WLD at foot-level (Table 9).

### 3.3. Associations with the Number of Feet Affected by White Line Disease per Ewe (Affected Sheep Only)

Univariable associations with the number of feet affected by WLD per ewe are presented in Supplementary Table S3. Four variables remained in the final multivariable model (Table 10). Ewes aged ≥4 years were also more likely to have more feet affected by WLD (β = 0.31, 95% CI: 0.15–0.47), compared to those aged <4 years. Ewes grazing a mix of permanent and new pasture were more likely to have more feet affected by WLD (β = 0.52, 95% CI: 0.12–0.92), than those grazing permanent pasture only. Furthermore, ewes were more likely to have less feet affected by WLD when grazing pasture at approximately >8 cm one month previously (β = −0.80, 95% CI: −1.22–−0.37), than when grazing shorter pasture (approximately 3 cm) one month previously. Ewes were more likely to have more feet affected by WLD at Visit 2 (β = 1.62, 95% CI: 1.29–1.95) and Visit 4 (β = 0.54, 95% CI: 0.31–0.76), but more likely to have less feet affected at Visit 3 (β = −0.48, 95% CI: −0.76–−0.19), compared to Visit 1.

**Figure 3:**
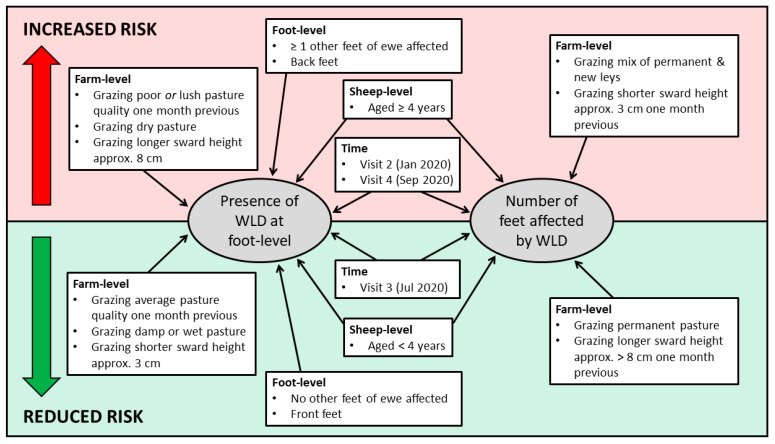
Summary of factors associated with the increased and reduced risk of white line disease at foot-level and the number of feet affected.

A summary of the risk factors for WLD presence at foot-level and number of feet affected per ewe is presented in Figure 3.

### 3.4. Associations with the Development of, and Recovery from, White Line Disease at Foot Level during Transition Period 1 (September 2019–January 2020)

#### 3.4.1. Development of White Line Disease during Transition Period 1

Univariable associations with the development of WLD at foot-level during T1 are presented in Supplementary Table S4. Three variables remained in the final multivariable model (Table 11). During T1, a foot had an increased risk of developing WLD if ≥1 other feet of the ewe also developed WLD. Furthermore, a foot had an increased risk of developing WLD if signs of FR were also present in the last month of the transition period (OR: 1.93, 95% CI: 1.20–3.09), compared to those with healthy feet. Also, feet of ewes in flocks with ≥500 ewes had a reduced risk of developing WLD (OR: 0.64, 95% CI: 0.47–0.87), compared to those in flocks with <500 ewes.

#### 3.4.2. Recovery from White Line Disease during Transition Period 1

Univariable associations with the recovery from WLD at foot-level during T1 are presented in Supplementary Table S5. Two variables remained in the final multivariable model (Table 12). During T1, a foot had an increased risk of recovering from WLD if ≥1 other feet of the ewe also recovered from WLD. Furthermore, the feet of ewes had an increased risk of recovering from WLD when the flock was vaccinated against FR (Footvax^®^) (OR: 1.64, 95% CI: 1.10–2.44), compared to flocks that were not vaccinated.

### 3.5. Associations with the Development of, and Recovery from, White Line Disease at Foot Level during Transition Period 2 (January 2020–July 2020)

#### 3.5.1. Development of White Line Disease during Transition Period 2

Univariable associations with the development of WLD at foot-level during T2 are presented in Supplementary Table S6. Two variables remained in the final multivariable model (Table 13). During T2, a foot had an increased risk of developing WLD if ≥1 other feet of the ewe also developed WLD. Also, feet of ewes had an increased risk of developing WLD when grazing a mixture of permanent and new pastures at the end of the transition period (OR: 3.22, 95% CI: 1.69–6.10), compared to those grazing permanent pasture only during this time.

#### 3.5.2. Recovery from White Line Disease during Transition Period 2

Univariable associations with the recovery from WLD at foot-level during T2 are presented in Supplementary Table S7. Three variables remained in the final multivariable model (Table 14). During T2, back feet had a reduced risk of recovering from WLD, compared to front feet (OR: 0.58, 95% CI: 0.42–0.80). A foot also had an increased risk of recovering from WLD if ≥1 other feet of the ewe also recovered from WLD. Furthermore, feet of ewes had a reduced risk of recovering from WLD when grazing both permanent and new pasture at the end of the transition period (OR: 0.38, 95% CI: 0.23–0.62), compared to permanent pasture only during this time.

### 3.6. Associations with the Development of, and Recovery from, White Line Disease at Foot Level during Transition Period 3 (July 2020–September 2020)

#### 3.6.1. Development of White Line Disease during Transition Period 3

Univariable associations with the development of WLD at foot-level during T3 are presented in Supplementary Table S8. Four variables remained in the final multivariable model (Table 15). During T3, back feet had an increased risk of developing WLD (OR: 1.83, 95% CI: 1.34–2.50). A foot also had an increased risk of developing WLD if ≥1 other feet of the ewe also developed WLD. Furthermore, a foot had an increased risk of developing WLD if signs of FR were also present at the last month of the transition period (OR: 1.81, 95% CI: 2.66–6.02), compared to healthy feet. Feet of ewes had a reduced risk of developing WLD when grazing poor pasture at the start of the transition period (OR: 0.60, 95% CI: 0.41–0.87), compared to those grazing lush pasture during this time.

#### 3.6.2. Recovery from White Line Disease during Transition Period 3

Univariable associations with the recovery from WLD at foot-level during T3 are presented in Supplementary Table S9. Three variables remained in the final multivariable model (Table 16). During T3, a foot had an increased risk of recovering from WLD if ≥1 other feet of the ewe also recovered from WLD. Furthermore, a foot had a reduced risk of recovering from WLD if signs of FR were present at the last month of the transition period (OR: 0.39, 95% CI: 0.17–0.89), compared to healthy feet. Feet of ewes also had a reduced risk of recovering from WLD when grazing a mixture of permanent and new pasture at the start of the transition period (OR: 0.45, 95% CI: 0.23–0.90), compared to those grazing permanent pasture only during this time.

**Figure 4:**
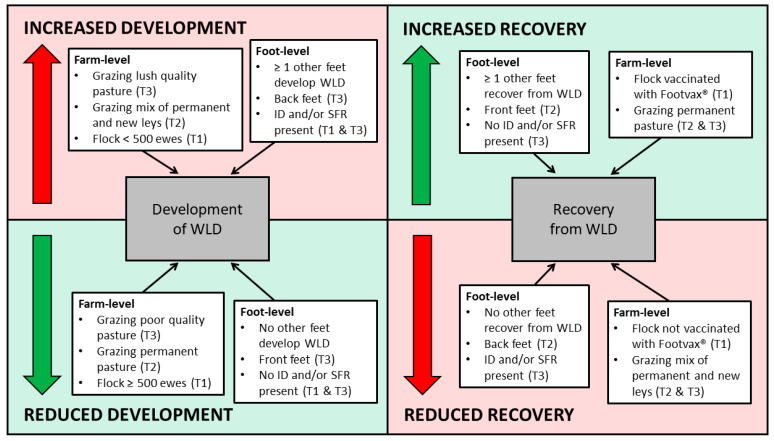
Summary of factors associated with the increased and reduced development of white line disease, and increased and reduced recovery from white line disease, at foot-level, and the number of feet affected. Brackets indicate the specific transition period.

A summary of the risk factors for the development of, and recovery from, WLD at foot-level is presented in Figure 4.

## 4. Discussion

This is the first study that presents novel, detailed data on the prevalence and patterns of WLD in commercial sheep flocks, and sheds new light on the transient and dynamic nature of WLD at foot-level. Our findings highlight the multifactorial causes of WLD, identifying key sheep, management and environmental factors at play, and advances our understanding of the aetiopathogenesis of WLD, whilst informing hypotheses for future prospective studies.

We highlight WLD as a significant, persistent problem affecting the feet of commercial sheep today, with little improvement in its prevalence in flocks since previous reports [10,19,25]. In our study, only 1% of ewes were not affected by WLD, with almost three-quarters of sheep-level observations and half of foot-level observations affected by WLD. Interestingly, back feet were most affected by WLD, and were most likely to have both paired claws affected, compared to front feet. This is in contrast to previous findings which have either highlighted a higher proportion of WLD in front feet [25], or no difference in prevalence by foot position [17], and the reasons behind these discrepancies are unclear. However, our findings align with that of dairy cattle; back feet of cattle are at higher risk of claw disorders and lameness, than front feet [28]. This is biologically plausible in cattle considering back feet are typically exposed to higher moisture levels and deeper faecal material [29], implicated as risk factors for WLD [30]. Hoof horn of back feet also have reduced puncture resistance to mechanical damage and foreign body penetration, compared to front feet [12]. We document back lateral claws to be most affected by WLD compared to other claw positions, further supporting observations of WLD in dairy cattle [31,32]. Greater vertical pressure is exerted on back lateral claws than medial claws in cattle [33], and assumed to be similar in sheep, which is likely to increase mechanical damage to hoof integrity and subsequent risk of clinical disorders. However, associations between body weight distribution or biomechanics and the prevalence of WLD have been previously contested in dairy ewes [17].

Whilst there is some observed foot-level heterogeneity in WLD presence, our findings suggest that there is a lack of independence in WLD occurrence and transition states at foot-level. Accordingly, the prevalence of WLD appears to be a problem at sheep-level. However, sheep-heterogeneity in WLD occurrence also occurred; a small number of sheep were never affected by WLD, whilst over one-third of sheep were persistently affected. This could explain the within-farm variation in WLD observed. In the present study, we identified feet of ewes aged ≥4 years to be more likely to have WLD present, and these ewes were more likely to have multiple feet affected, compared to younger ewes. Whilst no association between dairy ewe age and WLD was reported previously [17], our findings support studies in cattle reporting increasing age or parity to increase susceptibility to WLD [30,34,35]. Additionally, older ewes may be more prone to nutritional imbalance, resulting in the reduced production of good quality hoof tissue and increased subsequent risk of white line damage. Recurrent episodes of laminitis throughout a ewe’s lifetime may also increase susceptibility to WLD, due to chronic vascular injury of the hoof corium [36]. Further work into the nutritional profile of ewes, and the prevalence of laminitis, could help elucidate the key underlying mechanisms of WLD development and inform management strategies to reduce its prevalence.

We present for the first time the transient changes in WLD occurrence at foot-level. This has important implications on our understanding of the aetiopathogenesis of WLD. Firstly, feet can readily develop WLD within as little as two months, and this was the most frequent transition observed in our study. Secondly, the feet of sheep can readily recover from WLD within the short-term. Although damaged white line is weaker and at risk of prolonged, chronic impairment, resolution of WLD cavities could be facilitated by the higher rate of horn turnover in the white line [9]. This could explain why the majority of feet affected by WLD recovered, but some sheep never recovered from WLD. We postulate that inherent host factors may be responsible, or that chronic damage to the white line caused by WLD is irreversible in some instances. Due to the nature of the study, we cannot ascertain whether recurrent WLD from earlier years impacts mature ewes’ on-going susceptibility to WLD. Furthermore, we do not know the effect multiple short-term transitions from healthy to WLD, and WLD to healthy, have on the subsequent strength and integrity of the white line. Whilst we considered the use of a simplified, binary scoring system as optimal in exploring the prevalence and dynamics of WLD, scoring lesion severity could provide further insight into the pathogenesis of early and advanced lesions. It may be the case that smaller, discrete lesions are more likely to recover over the short-term than larger cavities.

A significant variation in the prevalence of WLD occurred over the four farms over time, and this was also reflected in our transition models. Feet were more likely to have WLD present at visit 2 (January 2020) and 4 (September 2020), and sheep were also more likely to have more feet affected at these time points, compared to visit 1 (September 2019). Furthermore, we demonstrate that feet were more likely to develop WLD during T1 (September 2019 to January 2020), than to remain healthy. Although it is difficult to separate the effects of lambing and indoor housing, and resultant changes to underfoot conditions, disruptions to hoof horn production and deterioration of the white line may occur around the time of parturition, similar to reports in cattle [37]. Feeding of concentrates during late gestation and early lactation may also increase susceptibility to subacute ruminal acidosis, laminitis and WLD [21]. In goats, ad libitum high-grain diets alter the rumen bacterial community and patterns of fermentation, which may contribute to laminar corium damage [38].

We found that feet were less likely to have WLD present at visit 3 (July 2020) than visit 1 and were more likely to recover from WLD during T2 (January 2020 to July 2020), than remain affected with WLD. This is a surprising finding, considering this period is assumed to be nutritionally demanding on ewes, and the impacts of lactation and nutrient partitioning could have an antagonistic effect on hoof tissue production. However, in cattle, the risk of WLD reduces in late lactation [39]. It may be possible that the feet experienced multiple transitions in WLD states during T2, due to this period spanning six months.

Interestingly, the prevalence of WLD in September 2020 did not return to levels observed in September 2019, suggesting that there is between-year variation in WLD prevalence. The variation in climatic conditions, and resultant pasture conditions, between years could explain this finding. The study period was defined by some atypical weather events; winter 2019 to early-spring 2020 was particularly mild and wet, and was followed by a period of unprecedented warm, dry weather with below average rainfall. This could lead to a seasonal ‘carry-over’ effect.

Our findings highlight co-infection with FR as a risk factor increasing the development of WLD. To the authors’ knowledge, this is the first time FR has been implicated in the development of non-infectious foot lesions, with comments previously highlighting WLD to predispose feet to FR [10]. Interestingly, we also document that feet of sheep in flocks vaccinated against FR (Footvax^®^, MSD Animal Health) were more likely to recover from WLD during T1. Although the biological reasons are unknown, we propose a number of explanations to these findings. One possibility is that the presence of *D. nodosus* on FR-affected feet may alter hoof horn growth [40], disrupting the structural integrity of the white line. Vaccinated flocks may have a lower prevalence of FR and circulating *D. nodosus*, particularly during the high-risk winter period, thus promoting WLD lesion resolution. A further explanation could be that FR and WLD share similar environmental and nutritional risk factors or genetic markers responsible for disease development. Although this requires further investigation through controlled experimental studies, reducing prevalence of infectious lameness could provide resistance to WLD development.

A number of pasture-based factors were identified to increase the risk of WLD presence on feet. Ewes grazing damp and wet pasture had a reduced risk of WLD, compared to those grazing dry pasture. However, neither rainfall nor temperature were retained as significant variables in final models. Hard, dry ground conditions, from higher daily temperatures, were previously associated with the shortening of hoof horn through greater wear [40]. The removal of the wall horn, through greater wearing forces, could increase contact between the sole and the ground, increasing risk of foreign body penetration and trauma to the white line. However, moisture was previously implicated in the loss of the structural integrity of the hoof horn [41] and increased the risk of WLD in cattle [35], which was assumed to be a similar in sheep. However, sheep are not typically exposed to slurry, unlike cattle, which could explain differences observed.

Longer swards also increased risk of WLD. These swards typically contain coarse, dense or stalky grass, and could increase abrasion to the feet of ewes, manifesting in white line deterioration. However, longer sward height was associated with fewer numbers of feet affected at sheep-level, and it is not clear why these results are contradictory. Previous studies have found that longer swards are associated with higher FR prevalence [42], and overall lameness prevalence [43], due to these pastures retaining greater moisture and humidity levels. However, in our study, pasture moisture had a negative association with WLD. Nonetheless, the high content of rye grasses found in long swards aligns with our finding that lush pasture quality increases risk of WLD, compared to average quality, and that lush pasture quality increased risk of WLD development during T3, compared to poor pasture. Dense, lush pastures have previously been implicated in the development of FR [44], and risk of CODD [25]. Lush pastures are also typically high in oligofructose, which when administered experimentally can induce laminar inflammatory responses and acute laminitis in sheep [45], and cattle [46].

Interestingly, ewes grazing a combination of permanent and new leys had an increased risk of having more feet affected by WLD, compared to those grazing permanent pasture only. Furthermore, grazing these pasture types also increased risk of WLD development during T2, and reduced WLD recovery during T2 and T3. Whilst these findings are difficult to explain due to the insufficient sample size of ewes grazing new leys alone, we speculate that the strong, coarse stems of grass species in new leys, relative to mixed species in permanent pastures, increases abrasive forces to the white line. Furthermore, grazing a variation of pasture types could cause fluctuations in rumen pH, impacting ruminal microbiota and increasing susceptibility to acidosis and laminitis [45].

Farm differences in WLD prevalence were observed, with Farm D having the highest WLD prevalence at foot-level and highest average number of feet affected per ewe, and Farm C having the lowest WLD prevalence and average number of feet affected per ewe. Interestingly, these two farms shared similar pasture conditions and soil type. This suggests that foot or sheep factors previously discussed have a greater impact on the susceptibility to WLD, or that between-flock variation is driven by management factors at farm-level. Other factors not considered in data collection, such as nutrition (composition of concentrates, supplements, forage, grass), and stocking density, could also explain between-farm differences observed. The effect of breed could not be estimated as was confounded by farm.

As with any longitudinal field survey, there are limitations that may affect interpretation of results. This was a study of four commercial sheep flocks, and as a result, the generalisability of our data is impacted by the particular farms chosen and findings should be interpreted with caution. Recall bias, farmers’ interpretation and resultant observer bias could impact estimates of the pasture variables obtained, and whilst this data is important, it is a very crude measure, and within-month variation will occur. More frequent scoring of pasture conditions, by a single observer, is warranted in future studies. Furthermore, objective measurements of soil temperature and moisture could provide greater detail of underfoot conditions at different time points on each farm. Unobserved differences between flocks were controlled for by including farm as a random effect in all models. Additionally, due to the nature of the study, the direction of causality of the associations between risk factors and WLD cannot be determined, although the findings do provide insight into the various factors at play. We were also unable to generate precise estimates of the average length of time sheep spent in each WLD state. We recognise that multiple transitions in WLD states could occur within the transition periods, particularly T2, and propose further prospective investigation in order to fully determine the time frames for WLD lesion development and resolution. Further studies could intensively observe sheep, with higher frequencies of uniform sampling points, to achieve data suitable for multilevel multistate discrete time event history models, which would prevent the fitting of separate models for each transition period. This would also account for the correlation between transitions, elucidating whether the duration of an episode of WLD affected subsequent risk of recovery. Furthermore, inspecting feet for clinical signs of chronic laminitis may prove useful in understanding its impact on WLD.

## 5. Conclusions

The results from our exploratory study provide key insight into the prevalence and dynamics of WLD in commercial sheep flocks over a 12-month period. Our findings highlight WLD to continue to persistently affect UK flocks, with feet undergoing a perpetual cycle of development, recovery and reoccurrence. We confirm that WLD is a complex, multifactorial hoof disorder and shed light on the wide-ranging factors associated with the aetiopathogenesis of WLD. Further prospective investigation is required to fully define the causality of associations between foot-, sheep- and farm-level factors and WLD prevalence and dynamics identified in this study. Breaking the dynamic cycle, to reduce WLD development, will prove important in reducing WLD prevalence. Given the high prevalence of WLD and the associated welfare implications, we believe further work is urgently required and justified as part of a concerted effort to reduce lameness in sheep.

## Figures and Tables

**Table 1:** Description of the four commercial sheep farms (A–D) selected for the study.

Farm	Location ^1^	Enterprises	Flock Size ^2^	System	Soil Type
A	Wales	Sheep, beef	500	Lowland	Loamy/clay mix
B	South West	Sheep, dairy	250	Lowland	Loamy
C	South West	Sheep, beef	540	Lowland	Clay
D	West Midlands	Sheep, arable	500	Lowland	Clay

^1^ UK region; ^2^ Number of breeding ewes.

**Table 2:** Distribution of ewes (*n* = 400) by farm and sampling frequency.

Farm	Total Ewes	Sampling Frequency		
		2	3	4
A	98	8	13	77
B	101	31	1	69
C	101	22	9	70
D	100	8	21	71
Total	400	69	44	287

**Table 3:** Description of variables considered when investigating associations with presence of white line disease at foot-level and number of feet affected at sheep-level.

Variable	Type	Description and Coding
*Sheep-level variables*		
Age	Categorical	Age of ewe at start of study 1 = <4 years 2 = ≥4 years
BCS	Categorical	Body condition score of ewe at time of visit 1 = 3.0 2 = <3.0 3 = >3.0
*Foot-level variables*		
Foot position	Categorical	1 = Front 2 = Back
Other feet affected by WLD	Categorical	Number of other feet of ewe affected by white line disease 0 = No other feet affected 1 = One other foot affected 2 = Two other feet affected 3 = Three other feet affected
Clinical disease	Categorical	Presence of FR on the foot 0 = No FR disease present 1 = ID and/or SFR present
*Farm-level variables*		
Flock size	Categorical	1 = <500 ewes 2 = ≥500 ewes
Vaccination status	Categorical	0 = Flock not vaccinated against footrot (Footvax^®^) 1 = Flock vaccinated against footrot (Footvax^®^)
Soil type	Categorical	1 = Loamy 2 = Clay 3 = Loamy/clay mix
Pasture moisture ^1^	Categorical	Average moisture of pasture grazed by ewes for calendar month 1 = Dry; hard ground, with little to no surface moisture 2 = Damp; firm ground, with moisture evident 3 = Wet; squelchy ground, but bears weight 4 = Saturated; boggy ground and bears no weight
Pasture quality ^1^	Categorical	Average quality of pasture grazed by ewes for calendar month 1 = Lush; approx. 80% rye grasses, mostly leaf 2 = Average; approx. 50% rye grasses, some stalk 3 = Poor; mostly stalk and weeds
Pasture type ^1^	Categorical	Average type of pasture grazed by ewes for calendar month 1 = Permanent grassland 2 = New grass ley 3 = Mix permanent and new ley
Sward height ^1^	Categorical	Average sward height of pasture grazed by ewes for calendar month 1 = Approx. 3 cm 2 = Approx. 8 cm 3 = Approx. >8 cm
Rainfall ^1^	Continuous	Average rainfall (mm) for calendar month, extracted from local MET Office data
Temperature ^1^	Continuous	Average maximum temperature (°C) for calendar month, extracted from local MET Office data
*Time variable*		
Visit	Categorical	Sampling visit number 1 = September 2019 2 = January 2020 3 = July 2020 4 = September 2020

^1^ Two separate variables considered in analyses; variable for the calendar month of visit and variable lagged to the previous calendar month.

**Table 4:** Description of variables considered when investigating associations with transitions in states of white line disease at foot-level.

Variable	Type	Description and Coding
*Sheep-level variables*		
Age	Categorical	Age of ewe at start of study 1 = <4 years 2 = ≥4 years
Change in BCS	Categorical	Change in body condition score of ewe during transition period 1 = Same condition 2 = Gained condition 3 = Lost condition
*Foot-level variables*		
Foot position	Categorical	1 = Front 2 = Back
Other feet developed WLD	Categorical	Number of other feet of ewe which developed white line disease 0 = None 1 = One other 2 = Two others 3 = Three others
Other feet recovered from WLD	Categorical	Number of other feet of ewe which recovered from white line disease 0 = None 1 = One other 2 = Two others 3 = Three others
Clinical disease ^1^	Categorical	Presence of FR on the foot 0 = No FR disease present 1 = ID and/or SFR present
*Farm-level variables*		
Flock size	Categorical	1 = <500 ewes 2 = ≥500 ewes
Vaccination status	Categorical	0 = Flock not vaccinated against footrot (Footvax^®^) 1 = Flock vaccinated against footrot (Footvax^®^)
Soil type	Categorical	1 = Loamy 2 = Clay 3 = Loamy/clay mix
Pasture moisture ^1^	Categorical	Average moisture of pasture grazed by ewes for calendar month 1 = Dry; hard ground, with little to no surface moisture 2 = Damp; firm ground, with moisture evident 3 = Wet; squelchy ground, but bears weight 4 = Saturated; boggy ground and bears no weight
Pasture quality ^1^	Categorical	Average quality of pasture grazed by ewes for calendar month 1 = Lush; approx. 80% rye grasses, mostly leaf 2 = Average; approx. 50% rye grasses, some stalk 3 = Poor; mostly stalk and weeds
Pasture type ^1^	Categorical	Average type of pasture grazed by ewes for calendar month 1 = Permanent grassland 2 = New grass ley 3 = Mix permanent and new ley
Sward height ^1^	Categorical	Average sward height of pasture grazed by ewes for calendar month 1 = Approx. 3 cm 2 = Approx. 8 cm 3 = Approx. >8 cm
Rainfall ^1^	Continuous	Average rainfall (mm) for calendar month, extracted from local MET Office data
Temperature ^1^	Continuous	Average maximum temperature (°C) for calendar month, extracted from local MET Office data

^1^ Two separate variables considered in the analyses: variable for the first calendar month and last calendar month of the transition period.

**Table 5:** Distribution of white line disease for 5672 foot-level observations of 400 ewes.

	Total Foot-Level Observations	WLD Present	
	*n*	*n*	%
All feet	5672	2657	46.8
Foot position			
Front	2836	1250	44.1
Back	2836	1407	49.6
Claw position			
Front lateral	1418	647	40.4
Front medial	1418	726	45.4
Back lateral	1418	769	48.1
Back medial	1418	720	45.0
Farm			
A	1452	720	49.6
B	1364	639	46.8
C	1404	525	37.4
D	1452	773	53.2
Visit			
1 (September 2019)	1556	526	33.8
2 (January 2020)	1536	898	58.5
3 (July 2020)	1356	449	33.1
4 (September 2020)	1224	784	64.1

**Table 6:** Number of feet per ewe affected by white line disease for 1086 sheep-level observations of 396 ewes affected.

	Total Sheep-Level Observations *n*	Number (%) of Feet Affected Per Ewe			
		1	2	3	4
All sheep	1086	272 (25.0)	317 (29.2)	237 (21.8)	260 (23.9)
Farm					
A	291	71 (24.4)	77 (26.5)	77 (26.5)	66 (22.7)
B	250	57 (22.8)	69 (27.6)	52 (20.8)	72 (28.8)
C	237	69 (29.1)	83 (35.0)	50 (21.1)	35 (14.8)
D	308	75 (28.6)	88 (28.6)	58 (18.8)	87 (28.2)
Visit					
1 (September 2019)	263	112 (42.6)	72 (27.4)	46 (17.5)	33 (12.5)
2 (January 2020)	329	47 (14.3)	95 (28.9)	87 (26.4)	100 (30.4)
3 (July 2020)	225	86 (38.2)	75 (33.3)	43 (19.1)	21 (9.3)
4 (September 2020)	269	27 (10.0)	75 (27.9)	61 (22.7)	106 (39.4)

**Table 7:** Distribution of ewes (*n* = 400) by farm and transition period.

Farm	Total Ewes *n*	Transition Period		
		T1	T2	T3
A	98	97	89	78
B	101	93	70	76
C	101	98	78	73
D	100	94	86	77
Total	400	382	323	304

T1: September 2019–January 2020; T2: January 2020–July 2020; T3: July 2020–September 2020.

**Table 8:** Distribution of white line disease transition states across three transition periods for 4036 foot-level observations of 400 ewes.

Transition State	Total Foot-Level Observations *n*	Transition Period					
		T1 (*n* = 1528)		T2 (*n* = 1292)		T3 (*n* = 1216)	
		*n*	%	*n*	%	*n*	%
Original state = healthy							
Remain healthy	1128	443	43.7	373	70.4	312	38.3
Develop WLD	1231	571	56.3	157	29.6	503	61.7
Original state = WLD							
Remain with WLD	855	320	62.3	260	34.1	275	68.6
Recover from WLD	822	194	37.7	502	65.9	126	31.4

T1: September 2019–January 2020; T2: January 2020–July 2020; T3: July 2020–September 2020.

**Table 9:** Final multivariable model of the associations with the presence of WLD for 5672 foot-level observations of 400 ewes.

Variable	*n*	%	Odds Ratio	Lower 95% CI	Upper 95% CI
Intercept			**0.36**	0.24	0.54
*Fixed effects*					
Age					
<4 years	3528	62.2	ref		
≥4 years	2144	37.8	**1.39**	1.19	1.62
Foot position					
Front	2836	50.0	ref		
Back	2836	50.0	**1.44**	1.26	1.64
Other feet affected by WLD					
None	1372	24.2	ref		
One other	1844	32.5	**2.47**	2.04	3.00
Two others	1535	27.1	**3.53**	2.90	4.28
Three others	921	16.2	**7.89**	6.29	9.88
Pasture moisture (calendar month of visit) (*n* = 4512)					
Dry (“hard”)	1008	22.3	ref		
Damp (“firm”)	752	16.7	**0.59**	0.44	0.79
Wet (“squelchy”)	2752	61.0	**0.52**	0.36	0.75
Saturated (“boggy”)	0	0.0	-	-	-
Pasture quality (lagged to previous calendar month)					
Lush (~90% leafy rye grasses)	2516	44.4	ref		
Average (~50% rye grasses)	2760	48.7	**0.50**	0.42	0.60
Poor (mostly stalk and weed)	396	7.0	**2.53**	1.78	3.60
Sward height (calendar month of visit) (*n* = 4512)					
Approx. 3 cm	1876	41.6	ref		
Approx. 8 cm	2324	51.5	**1.55**	1.09	2.22
Approx. > 8 cm	312	6.9	1.34	0.80	2.30
Visit					
1 (September 2019)	1556	27.4	ref		
2 (January 2020)	1536	27.1	**3.99**	2.44	6.53
3 (July 2020)	1356	23.9	**0.41**	0.27	0.63
4 (September 2020)	1224	21.6	**1.87**	1.24	2.83
*Random terms*	*Variance*	*SD*			
Ewe	<0.001	<0.001			
Farm	<0.001	<0.001			

CI: confidence interval for odds ratio; bold odds ratios are statistically significant at 0.05 as their CIs do not include 1; ref: baseline category for comparison; SD: standard deviation.

**Table 10:** Final multivariable model of the associations with the number of feet affected by white line disease for 1086 sheep-level observations of 396 ewes affected.

Variable	*n*	%	β	Lower 95% CI	Upper 95% CI
Intercept			**1.90**	1.40	2.40
*Fixed effects*					
Age					
<4 years	672	61.9	ref		
≥4 years	414	38.1	**0.31**	0.15	0.47
Pasture type (calendar month of visit) (*n* = 838)					
Permanent grassland	149	17.8	ref		
New grass ley	135	16.1	−0.41	-1.03	0.22
Mix permanent and new ley	554	66.1	**0.52**	0.12	0.92
Sward height (lagged to previous calendar month)					
Approx. 3 cm	400	36.8	ref		
Approx. 8 cm	608	56.0	−0.04	−0.43	0.35
Approx. >8 cm	78	7.2	**−0.80**	−1.22	−0.37
Visit					
1 (September 2019)	263	24.2	ref		
2 (January 2020)	329	30.3	**1.62**	1.29	1.95
3 (July 2020)	225	20.7	**−0.48**	−0.76	−0.19
4 (September 2020)	269	24.8	**0.54**	0.31	0.76
*Random terms*	*Variance*	*SD*			
Ewe	0.04	0.20			
Farm	0.05	0.22			

β: coefficient; CI: confidence interval for coefficient; bold coefficients are statistically significant at 0.05 as their CIs do not include 0; ref: baseline category for comparison; SD: standard deviation.

**Table 11:** Final multivariable model of the associations with the development of WLD at foot-level for 1014 observations of 351 ewes during transition period 1 (September 2019–January 2020).

Variable	*n*	%	Odds Ratio	Lower 95% CI	Upper 95% CI
Intercept			**0.67**	0.47	0.94
*Fixed effects*					
Other feet develop WLD					
None	284	28.0	ref		
One other	308	30.4	**2.33**	1.67	3.26
Two others	278	27.4	**4.77**	3.32	6.84
Three others	144	14.2	**6.13**	3.83	9.81
Clinical disease (last month of T1)					
No FR disease present	914	90.1	ref		
ID and/or SFR present	100	9.9	**1.93**	1.20	3.09
Flock size					
<500 ewes	289	28.5	ref		
≥500 ewes	725	71.5	**0.64**	0.47	0.87
*Random terms*	*Variance*	*SD*			
Ewe	<0.001	<0.001			
Farm	<0.001	<0.001			

CI: confidence interval for odds ratio; bold odds ratios are statistically significant at 0.05 as their CIs do not include 1; ref: baseline category for comparison; SD: standard deviation.

**Table 12:** Final multivariable model of the associations with the recovery from WLD at foot-level for 514 observations of 256 ewes during transition period 1 (September 2019–January 2020).

Variable	*n*	%	Odds Ratio	Lower 95% CI	Upper 95% CI
Intercept			**0.29**	0.21	0.41
*Fixed effects*					
Other feet recover from WLD					
None	309	60.1	Ref		
One other	119	23.2	**1.89**	1.21	2.96
Two others	65	12.6	**4.16**	2.33	7.45
Three others	21	4.1	**6.98**	2.45	19.92
Vaccination status					
Flock not vaccinated with Footvax^®^	224	43.6	Ref		
Flock vaccinated with Footvax^®^	290	56.4	**1.64**	1.10	2.44
*Random terms*	*Variance*	*SD*			
Ewe	<0.001	<0.001			
Farm	<0.001	<0.001			

CI: confidence interval for odds ratio; bold odds ratios are statistically significant at 0.05 as their CIs do not include 1; ref: baseline category for comparison; SD: standard deviation.

**Table 13:** Final multivariable model of the associations with the development of WLD at foot-level for 530 observations of 235 ewes during transition period 2 (January 2020–July 2020).

Variable	*n*	%	Odds Ratio	Lower 95% CI	Upper 95% CI
Intercept			**0.09**	0.05	0.17
*Fixed effects*					
Other feet develop WLD					
None	332	62.6	ref		
One other	145	27.4	**2.26**	1.45	3.53
Two others	42	7.9	**2.15**	1.08	4.28
Three others	11	2.1	**7.45**	1.89	29.37
Pasture type (last month of T2)					
Permanent grassland	144	27.2	ref		
New grass ley	0	0.0	-	-	-
Mix permanent and new ley	386	72.8	**3.22**	1.69	6.10
*Random terms*	*Variance*	*SD*			
Ewe	<0.001	<0.001			
Farm	0.017	0.129			

CI: confidence interval for odds ratio; bold odds ratios are statistically significant at 0.05 as their CIs do not include 1; ref: baseline category for comparison; SD: standard deviation.

**Table 14:** Final multivariable model of the associations with the recovery from WLD at foot-level for 762 observations of 275 ewes during transition period 2 (January 2020–July 2020).

Variable	*n*	%	Odds Ratio	Lower 95% CI	Upper 95% CI
Intercept			**2.47**	1.43	4.28
*Fixed effects*					
Foot position					
Front	382	50.1	ref		
Back	380	49.9	**0.58**	0.42	0.80
Other feet recover from WLD					
None	168	22.0	ref		
One other	241	31.6	**2.14**	1.41	3.24
Two others	214	28.1	**2.88**	1.86	4.47
Three others	139	18.2	**6.36**	3.65	11.08
Pasture type (last month of T2)					
Permanent grassland	168	22.0	ref		
New grass ley	0	0.0	-	-	-
Mix permanent and new ley	594	78.0	**0.38**	0.23	0.62
*Random terms*	*Variance*	*SD*			
Ewe	<0.001	<0.001			
Farm	0.010	0.102			

CI: confidence interval for odds ratio; bold odds ratios are statistically significant at 0.05 as their CIs do not include 1; ref: baseline category for comparison; SD: standard deviation.

**Table 15:** Final multivariable model of the associations with the development of WLD at foot-level for 815 observations of 286 ewes during transition period 3 (July 2020–September 2020).

Variable	*n*	%	Odds Ratio	Lower 95% CI	Upper 95% CI
Intercept			**0.44**	0.30	0.64
*Fixed effects*					
Foot position					
Front	380	46.6	Ref		
Back	435	53.4	**1.83**	1.34	2.50
Other feet develop WLD					
None	204	25.0	Ref		
One other	233	28.6	**4.00**	2.66	6.02
Two others	237	29.1	**3.62**	2.41	5.44
Three others	141	17.3	**11.46**	6.54	20.08
Clinical disease (last month of T3)					
No FR disease present	727	89.2	Ref		
ID and/or SFR present	88	10.8	**1.81**	2.66	6.02
Pasture quality (first month of T3)					
Lush (~90% leafy rye grasses)	364	44.7	Ref		
Average (~50% rye grasses)	208	25.5	0.98	0.65	1.47
Poor (mostly stalk and weed)	243	29.8	**0.60**	0.41	0.87
*Random terms*	*Variance*	*SD*			
Ewe	<0.001	<0.001			
Farm	<0.001	<0.001			

CI: confidence interval for odds ratio; bold odds ratios are statistically significant at 0.05 as their CIs do not include 1; ref: baseline category for comparison; SD: standard deviation.

**Table 16:** Final multivariable model of the associations with the recovery from WLD at foot-level for 401 observations of 200 ewes during transition period 3 (July 2020–September 2020).

Variable	*n*	%	Odds Ratio	Lower 95% CI	Upper 95% CI
Intercept			**0.50**	0.26	0.96
*Fixed effects*					
Other feet recover from WLD					
None	274	68.3	ref		
One other	82	20.4	**6.06**	3.51	10.47
Two others	34	8.5	**6.23**	2.83	13.69
Three others	11	2.7	**11.82**	3.00	46.53
Clinical disease (last month of T3)					
No FR disease present	339	84.5	ref		
ID and/or SFR present	62	15.5	**0.39**	0.17	0.89
Pasture type (first month of T3)					
Permanent grassland	49	12.2	ref		
New grass ley	0	0.0	-	-	-
Mix permanent and new ley	352	87.8	**0.45**	0.23	0.90
*Random terms*	*Variance*	*SD*			
Ewe	<0.001	<0.001			
Farm	<0.001	<0.001			

CI: confidence interval for odds ratio; bold odds ratios are statistically significant at 0.05 as their CIs do not include 1; ref: baseline category for comparison; SD: standard deviation.

## Data Availability

The data presented in this study are available on reasonable request from the corresponding author. The data is not publicly available as not all data from the study has been published yet.

## Supplementary Materials

The following are available online at https://www.mdpi.com/article/10.3390/vetsci8060116/s1, Table S1: Location of local MET Office weather stations for each farm in the study, Table S2: Univariable analyses of the associations with the presence of WLD for 5672 foot-level observations of 400 sheep, Table S3: Univariable analyses of the associations with the number of feet affected by WLD for 1086 sheep-level observations of 396 ewes affected by WLD, Table S4: Univariable analyses of the associations with the development of WLD at foot-level for 1014 observations of 351 ewes during transition period 1 (September 2019–January 2020), Table S5: Univariable analyses of the associations with the recovery from WLD at foot-level for 514 observations of 256 ewes during transition period 1 (September 2019–January 2020), Table S6: Univariable analyses of the associations with the development of WLD at foot-level for 530 observations of 235 ewes during transition period 2 (January 2020–July 2020), Table S7: Univariable analyses of the associations with the recovery from WLD at foot-level for 762 observations of 275 ewes during transition period 2 (January 2020–July 2020), Table S8: Univariable analyses of the associations with the development of WLD at foot-level for 815 observations of 286 ewes during transition period 3 (July 2020–September 2020), Table S9: Univariable analyses of the associations with the recovery from WLD at foot-level for 401 observations of 200 ewes during transition period 3 (July 2020–September 2020).
