# Supplementary material for: Prevalence and Temporal Dynamics of White Line Disease in Sheep: An Exploratory Investigation into Disease Distribution and Associated Risk Factors
Source: Vet Sci. 2021 Jun 19;8(6):116. doi: 10.3390/vetsci8060116 (PMC8234444; doi:10.3390/vetsci8060116)
Supplement: Supplementary file 1 [file vetsci-08-00116-s001.zip › vetsci-1258289-supplementary.pdf]

Supplementary Materials

Table S1. Location of local MET Office weather stations for each farm in the study.

Farm	Weather station location	Approximate distance from farm (km)
A	Cardiff Bute Park, Wales	11.7
B	Oxford, South East	26.0
C	Ross-on-Wye, West Midlands	12.7
D	Ross-on-Wye, West Midlands	23.8

Table S2. Univariable analyses of the associations with the presence of WLD for 5672 foot-level observations of 400 sheep.

Variable	*n*	%	Odds ratio	Lower 95% CI	Upper 95% CI	*p*
Sheep-level variables						
**Age**						
< 4 years	3528	62.2	ref			
≥ 4 years	2144	37.8	**1.36**	1.14	1.63	0.001
**BCS**						
3.0	2816	49.6	ref			
< 3.0	828	14.6	**0.66**	0.54	0.80	<0.001
> 3.0	2028	35.8	**1.28**	1.10	1.49	<0.001
Foot-level variables						
**Foot position**						
Front	2836	50.0	ref			
Back	2836	50.0	**1.27**	1.14	1.42	<0.001
**Other feet affected by WLD**						
None	1372	24.2	ref			
One other	1844	32.5	**2.86**	2.45	3.35	<0.001
Two others	1535	27.1	**4.07**	3.46	4.79	<0.001
Three others	921	16.2	**9.24**	7.60	11.25	<0.001
**Clinical disease**						
No FR disease present	5204	91.7	ref			
ID and/or SFR present	468	8.3	**2.05**	1.65	2.56	<0.001
Farm-level variables						
**Flock size**						
< 500 ewes	1364	24.0	ref			
≥ 500 ewes	4308	76.0	0.99	0.57	1.74	0.982
**Vaccination status**						
Flock not vaccinated against footrot (Footvax®)	2816	49.6	ref			
Flock vaccinated against footrot (Footvax®)	2856	50.4	0.76	0.51	1.13	0.174
**Soil type**						
Loamy	1364	24.0	ref			
Clay	2856	50.4	0.94	0.53	1.66	0.819
Loamy/clay mix	1452	25.6	1.12	0.58	2.16	0.736
**Pasture moisture (calendar month of visit) (*n* = 4512)**						
Dry (“hard”)	996	22.1	ref			
Damp (“firm”)	2064	45.7	**1.88**	1.59	2.23	<0.001

Wet (“squelchy”)	1452	32.2	**2.38**	1.92	2.94	<0.001
Saturated (“boggy”)	0	0.0	**-**	-	-	-
**Pasture moisture (lagged to previous calendar month)**						
Dry (“hard”)	1604	28.3	ref			
Damp (“firm”)	2140	37.7	0.86	0.74	1.00	0.054
Wet (“squelchy”)	768	13.5	**1.96**	1.59	2.42	<0.001
Saturated (“boggy”)	1160	20.5	**1.72**	1.45	2.03	<0.001
**Pasture quality (calendar month of visit) (*n* = 4512)**						
Lush (~ 90% leafy rye grasses)	2812	62.3	ref			
Average (~ 50% rye grasses)	1700	37.7	**1.23**	1.01	1.49	0.041
Poor (mostly stalk and weeds)	0	0.0	**-**	-	-	-
**Pasture quality (lagged to previous calendar month)**						
Lush (~ 90% leafy rye grasses)	2516	44.4	ref			
Average (~ 50% rye grasses)	2760	48.7	0.92	0.73	1.18	0.528
Poor (mostly stalk and weeds)	396	7.0	**2.53**	1.78	3.60	<0.001
**Pasture type (calendar month of visit) (*n* = 4512)**						
Permanent grassland	1008	22.3	ref			
New grass ley	752	16.7	**2.01**	1.56	2.60	<0.001
Mix permanent and new ley	2752	61.0	**2.30**	1.87	2.84	<0.001
**Pasture type (lagged to previous calendar month)**						
Permanent grassland	1768	31.2	ref			
New grass ley	3584	63.2	**0.66**	0.52	0.83	<0.001
Mix permanent and new ley	320	5.6	**0.23**	0.17	0.32	<0.001
**Sward height (calendar month of visit) (*n* = 4512)**						
Approx. 3 cm	1876	41.6	ref			
Approx. 8 cm	2324	51.5	**2.48**	2.15	2.86	<0.001
Approx. > 8 cm	312	6.9	**5.50**	4.04	7.48	<0.001
**Sward height (lagged to previous calendar month)**						
Approx. 3 cm	2068	36.5	ref			
Approx. 8 cm	3208	56.6	**1.19**	1.02	1.38	0.027
Approx. > 8 cm	396	7.0	**0.41**	0.32	0.53	<0.001
**Rainfall (calendar month of visit)**	4512	100.0	**0.99**	0.99	0.99	<0.001
**Rainfall (lagged to previous calendar month)**	5672	100.0	**1.03**	1.03	1.03	<0.001
**Temperature (calendar month of visit)**	4512	100.0	**0.87**	0.85	0.89	<0.001
**Temperature (lagged to previous calendar month)**	5672	100.0	**0.95**	0.94	0.96	<0.001

Time variable						
**Visit**						
1 (Sep 2019)	1556	27.4	ref			
2 (Jan 2020)	1536	27.1	**3.10**	2.66	3.62	<0.001
3 (Jul 2020)	1356	23.9	0.94	0.80	1.10	0.449
4 (Sep 2020)	1224	21.6	**4.00**	3.38	4.73	<0.001

CI: confidence interval for odds ratio; bold odds ratios are statistically significant at 0.05 as their CIs do not include 1; ref: baseline category for comparison.

Table S3. Univariable analyses of the associations with the number of feet affected by WLD for 1086 sheep-level observations of 396 ewes affected by WLD.

Variable	*n*	%	β	Lower 95% CI	Upper 95% CI	*p*
Sheep-level variables						
**Age**						0.001
< 4 years	672	61.9	ref			
≥ 4 years	414	38.1	**0.29**	0.11	0.46	
**BCS**[1]						0.005
3.0	523	48.2	ref			
< 3.0	149	13.7	**-0.26**	-0.49	-0.03	
> 3.0	414	38.1	0.18	0.00	0.37	
**Clinical disease**						<0.001
No feet with FR disease present	920	84.7	ref			
≥ 1 feet with ID and/or SFR present	166	15.3	**0.35**	0.16	0.54	
Farm-level variables						
**Flock size**						0.991
< 500 ewes	250	23.0	ref			
≥ 500 ewes	836	77.0	0.00	-0.75	0.75	
**Vaccination status**						0.341
Flock not vaccinated against footrot (Footvax®)	558	51.4	ref			
Flock vaccinated against footrot (Footvax®)	528	48.6	-0.26	-0.80	0.28	
**Soil type**						0.953
Loamy	545	50.2	ref			
Clay	250	23.0	0.06	-1.02	1.14	
Loamy/clay mix	291	26.8	0.11	-1.13	1.35	
**Pasture moisture (calendar month of visit) (*n* = 838)**						<0.001
Dry ("hard")	159	19.0	ref			
Damp ("firm")	394	47.0	**0.52**	0.31	0.73	
Wet ("squelchy")	285	34.0	**0.71**	0.45	0.98	
Saturated ("boggy")	0	0.0	-	-	-	-
**Pasture moisture (lagged to previous calendar month)**						<0.001
Dry ("hard")	293	27.0	ref			
Damp ("firm")	383	35.3	-0.15	-0.34	0.05	
Wet ("squelchy")	162	14.9	**0.59**	0.32	0.86	
Saturated ("boggy")	248	22.8	**0.47**	0.25	0.68	
**Pasture quality (calendar month of visit) (*n* = 838)**						0.176

Lush (~ 90% leafy rye grasses)	559	66.7	ref			
Average (~ 50% rye grasses)	279	33.3	0.17	-0.08	0.41	
Poor (mostly stalk and weeds)	0	0.0	-	-	-	-
**Pasture quality (lagged to previous calendar month)**						<0.001
Lush (~ 90% leafy rye grasses)	525	48.3	ref			
Average (~ 50% rye grasses)	473	43.6	-0.11	-0.41	0.19	
Poor (mostly stalk and weeds)	88	8.1	**0.77**	0.34	1.21	
**Pasture type (calendar month of visit) (*n* = 838)**						<0.001
Permanent grassland	149	17.8	ref			
New grass ley	135	16.1	**0.59**	0.32	0.85	
Mix permanent and new ley	554	66.1	**0.74**	0.54	0.94	
**Pasture type (lagged to previous calendar month)**						<0.001
Permanent grassland	335	30.8	ref			
New grass ley	718	66.1	**-0.36**	-0.64	-0.09	
Mix permanent and new ley	30	3.0	**-1.09**	-1.43	-0.75	
**Sward height (calendar month of visit) (*n* = 838)**						<0.001
Approx. 3 cm	296	35.3	ref			
Approx. 8 cm	474	56.6	**0.79**	0.62	0.96	
Approx. > 8 cm	68	8.1	**1.46**	1.11	1.81	
**Sward height (lagged to previous calendar month)**						<0.001
Approx. 3 cm	400	36.8	ref			
Approx. 8 cm	608	56.0	0.15	-0.05	0.34	
Approx. > 8 cm	78	7.2	**-0.80**	-1.13	-0.48	
**Rainfall (calendar month of visit)**	5672	100.0	**-0.01**	-0.01	-0.01	<0.001
**Rainfall (lagged to previous calendar month)**	5672	100.0	**-0.12**	-0.15	-0.09	<0.001
**Temperature (calendar month of visit)**	5672	100.0	**0.03**	0.02	0.03	<0.001
**Temperature (lagged to previous calendar month)**	5672	100.0	**-0.05**	-0.06	-0.04	<0.001
Time variable						
**Visit**						<0.001
1 (Sep 2019)	263	24.2	ref			
2 (Jan 2020)	329	30.3	**0.99**	0.81	1.17	
3 (Jul 2020)	225	20.7	-0.04	-0.22	0.14	
4 (Sep 2020)	269	24.8	**1.21**	1.02	1.40	

β: estimate; CI: confidence interval for estimate; bold estimates are statistically significant at 0.05 as their CIs do not include 0; ref: baseline category for comparison.

Table S4. Univariable analyses of the associations with the development of WLD at foot-level for 1014 observations of 351 ewes during transition period 1 (Sep 2019 – Jan 2020).

Variable	*n*	%	Odds ratio	Lower 95% CI	Upper 95% CI	*p*
Sheep-level variables						
**Age**						
< 4 years	661	65.2	ref			
≥ 4 years	353	34.8	**1.72**	1.04	2.83	0.033
**Change in BCS**						
Same condition	626	61.7	ref			
Gained condition	164	16.2	1.09	0.60	1.97	0.781
Lost condition	224	22.1	0.85	0.50	1.46	0.559
Foot-level variables						
**Foot position**						
Front	497	49.0	ref			
Back	517	51.0	1.13	0.84	1.51	0.429
**Other feet develop WLD**						
None	284	28.0	ref			
One other	308	30.4	**2.38**	1.70	3.33	<0.001
Two others	278	27.4	**4.85**	3.38	6.95	<0.001
Three others	144	14.2	**6.63**	4.14	10.62	<0.001
**Clinical disease (First month of T1)**						
No FR disease present	954	94.1	ref			
ID and/or SFR present	60	5.9	1.00	0.48	2.09	0.984
**Clinical disease (Last month of T1)**						
No FR disease present	914	90.1	ref			
ID and/or SFR present	100	9.9	**1.99**	1.07	3.71	0.031
Farm-level variables						
**Flock size**						
< 500 ewes	289	28.5	ref			
≥ 500 ewes	725	71.5	**0.42**	0.41	0.42	<0.001
**Vaccination status**						
Flock not vaccinated against footrot (Footvax®)	524	51.7	ref			
Flock vaccinated against footrot (Footvax®)	490	48.3	**0.46**	0.30	0.69	<0.001
**Soil type**						
Loamy	289	28.5	ref			
Clay	528	52.1	**2.21**	2.20	2.21	<0.001
Loamy/clay mix	197	19.4	**0.74**	0.74	0.74	<0.001

**Pasture moisture (First month of T1)**						
Dry (“hard”)	0	0.0	-	-	-	-
Damp (“firm”)	528	52.1	ref			
Wet (“squelchy”)	486	47.9	1.28	0.60	2.73	0.520
Saturated (“boggy”)	0	0.0	-	-	-	-
**Pasture quality (First month of T1)**						
Lush (~ 90% leafy rye grasses)	721	71.1	ref			
Average (~ 50% rye grasses)	293	28.9	0.66	0.29	1.48	0.311
Poor (mostly stalk and weeds)	0	0.0	-	-	-	-
**Pasture type (First month of T1)**						
Permanent grassland	293	28.9	ref			
New grass ley	289	28.5	**2.63**	2.62	2.64	<0.001
Mix permanent and new ley	432	42.6	**1.16**	1.15	1.16	<0.001
**Sward height (First month of T1)**						
Approx. 3 cm	293	28.9	ref			
Approx. 8 cm	721	71.1	1.52	0.68	3.39	0.311
Approx. > 8cm	0	0.0	-	-	-	-
**Rainfall (calendar month of visit)**	1014	100.0	1.00	0.99	1.01	0.053
**Temperature (calendar month of visit)**	1014	100.0	0.43	0.07	2.67	0.364

CI: confidence interval for odds ratio; bold odds ratios are statistically significant at 0.05 as their CIs do not include 1; ref: baseline category for comparison.

Table S5. Univariable analyses of the associations with the recovery from WLD at foot-level for 514 observations of 256 ewes during transition period 1 (Sep 2019 – Jan 2020).

**Variable**	***n***	**%**	**Odds ratio**	**Lower 95% CI**	**Upper 95% CI**	***p***
Sheep-level variables						
**Age**						
< 4 years	279	54.3	ref			
≥ 4 years	235	45.7	0.81	0.46	1.44	0.477
**Change in BCS**						
Same condition	290	56.4	ref			
Gained condition	72	14.0	1.16	0.52	2.58	0.709
Lost condition	152	29.6	0.75	0.38	1.48	0.408
Foot-level variables						
**Foot position**						
Front	267	51.9	ref			
Back	247	48.1	1.17	0.76	1.79	0.483
**Other feet recover from WLD**						
None	309	60.1	ref			
One other	119	23.2	**1.95**	1.24	3.07	0.004
Two others	65	12.6	**4.38**	2.42	7.94	<0.001
Three others	21	4.1	**7.25**	2.49	21.10	<0.001
**Clinical disease (First month of T1)**						
No FR disease present	467	90.9	ref			
ID and/or SFR present	47	9.1	1.39	0.59	3.25	0.447
**Clinical disease (Last month of T1)**						
No FR disease present	432	84.0	ref			
ID and/or SFR present	82	16.0	0.77	0.38	1.57	0.470
Farm-level variables						
**Flock size**						
< 500 ewes	83	16.1	ref			
≥ 500 ewes	431	83.9	1.83	0.61	5.47	0.283
**Vaccination status**						
Flock not vaccinated against footrot (Footvax®)	224	43.6	ref			
Flock vaccinated against footrot (Footvax®)	290	56.4	**2.77**	1.60	4.80	<0.001
**Soil type**						
Loamy	83	16.1	ref			
Clay	240	46.7	1.22	0.38	3.96	0.453
Loamy/clay mix	191	37.2	**2.94**	1.63	5.32	<0.001

**Pasture moisture (First month of T1)**						
Dry ("hard")	0	0.0	-	-	-	-
Damp ("firm")	240	46.7	ref			
Wet ("squelchy")	274	53.3	1.65	0.68	3.97	0.267
Saturated ("boggy")	0	0.0	-	-	-	-
**Pasture quality (First month of T1)**						
Lush (~ 90% leafy rye grasses)	415	80.7	ref			
Average (~ 50% rye grasses)	99	19.3	1.02	0.31	3.37	0.975
Poor (mostly stalk and weeds)	0	0.0	-	-	-	-
**Pasture type (First month of T1)**						
Permanent grassland	99	19.3	ref			
New grass ley	83	16.1	0.62	0.16	2.35	0.483
Mix permanent and new ley	332	64.6	1.20	0.40	3.62	0.750
**Sward height (First month of T1)**						
Approx. 3 cm	99	19.3	ref			
Approx. 8 cm	415	90.7	0.98	0.30	3.24	0.975
Approx. > 8cm	0	0.0	-	-	-	-
**Rainfall (calendar month of visit)**	514	100.0	**1.02**	1.01	1.02	<0.001
**Temperature (calendar month of visit)**	514	100.0	**10.02**	3.10	32.43	<0.001

CI: confidence interval for odds ratio; bold odds ratios are statistically significant at 0.05 as their CIs do not include 1; ref: baseline category for comparison.

Table S6. Univariable analyses of the associations with the development of WLD at foot-level for 530 observations of 235 ewes during transition period 2 (Jan 2020 – Jul 2020).

Variable	*n*	%	Odds ratio	Lower 95% CI	Upper 95% CI	*p*
Sheep-level variables						
**Age**						
< 4 years	354	66.8	ref			
≥ 4 years	176	33.2	0.81	0.46	1.43	0.465
**Change in BCS**						
Same condition	261	49.2	ref			
Gained condition	116	21.9	**0.44**	0.20	0.96	0.038
Lost condition	153	28.9	0.80	0.40	1.62	0.543
Foot-level variables						
**Foot position**						
Front	264	49.8	ref			
Back	266	50.2	**1.65**	1.06	2.54	0.025
**Other feet develop WLD**						
None	332	62.6	ref			
One other	145	27.4	**2.29**	1.48	3.56	<0.001
Two others	42	7.9	**2.27**	1.14	4.51	0.019
Three others	11	2.1	**7.58**	1.92	30.02	0.004
**Clinical disease (First month of T2)**						
No FR disease present	481	90.8	ref			
ID and/or SFR present	49	9.2	1.45	0.64	3.27	0.371
**Clinical disease (Last month of T2)**						
No FR disease present	523	98.7	ref			
ID and/or SFR present	7	1.3	3.58	0.50	25.68	0.204
Farm-level variables						
**Flock size**						
< 500 ewes	85	16.0	ref			
≥ 500 ewes	445	84.0	0.81	0.15	4.39	0.805
**Vaccination status**						
Flock not vaccinated against footrot (Footvax®)	206	38.9	ref			
Flock vaccinated against footrot (Footvax®)	324	61.1	0.35	0.12	1.01	0.053
**Soil type**						
Loamy	85	16.0	ref			
Clay	265	50.0	1.38	0.24	8.11	0.720
Loamy/clay mix	180	34.0	1.38	0.25	7.68	0.713

**Pasture moisture (Last month of T2)**						
Dry (“hard”)	0	0.0	-	-	-	-
Damp (“firm”)	350	66.0	ref			
Wet (“squelchy”)	180	34.0	1.25	0.24	6.50	0.792
Saturated (“boggy”)	0	0.0	-	-	-	-
**Pasture quality (Last month of T2)**						
Lush (~ 90% leafy rye grasses)	301	56.8	ref			
Average (~ 50% rye grasses)	85	16.0	0.73	0.35	1.49	0.383
Poor (mostly stalk and weeds)	144	27.2	**0.17**	0.08	0.36	<0.001
**Pasture type (Last month of T2)**						
Permanent grassland	144	27.2	ref			
New grass ley	0	0.0	-	-	-	-
Mix permanent and new ley	386	72.8	**5.41**	2.62	11.18	<0.001
**Sward height (Last month of T2)**						
Approx. 3 cm	206	38.9	ref			
Approx. 8 cm	324	61.1	0.35	0.12	1.01	0.053
Approx. > 8 cm	0	0.0	-	-	-	-
**Rainfall (calendar month of visit)**	530	100.0	1.00	0.97	1.04	0.880
**Temperature (calendar month of visit)**	530	100.0	2.16	0.02	200.44	0.739

CI: confidence interval for odds ratio; bold odds ratios are statistically significant at 0.05 as their CIs do not include 1; ref: baseline category for comparison.

Table S7. Univariable analyses of the associations with the recovery from WLD at foot-level for 762 observations of 275 ewes during transition period 2 (Jan 2020 – Jul 2020).

Variable	*n*	%	Odds ratio	Lower 95% CI	Upper 95% CI	*p*
Sheep-level variables						
**Age**						
< 4 years	458	60.1	ref			
≥ 4 years	304	39.9	1.18	0.71	1.97	0.530
**Change in BCS**						
Same condition	327	42.9	ref			
Gained condition	212	27.8	1.09	0.54	2.18	0.818
Lost condition	223	29.3	1.09	0.60	1.95	0.785
Foot-level variables						
**Foot position**						
Front	382	50.1	ref			
Back	380	49.9	**0.57**	0.40	0.81	0.002
**Other feet recover from WLD**						
None	168	22.0	ref			
One other	241	31.6	**2.04**	1.35	3.07	0.004
Two others	214	28.1	**2.69**	1.74	4.16	<0.001
Three others	139	18.2	**5.82**	3.36	10.07	<0.001
**Clinical disease (First month of T2)**						
No FR disease present	649	85.2	ref			
ID and/or SFR present	113	14.8	0.62	0.34	1.11	0.107
**Clinical disease (Last month of T2)**						
No FR disease present	747	98.0	ref			
ID and/or SFR present	15	2.0	0.32	0.08	1.39	0.129
Farm-level variables						
**Flock size**						
< 500 ewes	195	25.6	ref			
≥ 500 ewes	567	74.4	0.84	0.25	2.84	0.774
**Vaccination status**						
Flock not vaccinated against footrot (Footvax®)	418	54.9	ref			
Flock vaccinated against footrot (Footvax®)	344	45.1	1.88	0.78	4.52	0.159
**Soil type**						
Loamy	195	25.6	ref			
Clay	391	51.3	1.12	0.31	4.05	0.863
Loamy/clay mix	176	23.1	0.82	0.23	2.96	0.761

**Pasture moisture (Last month of T2)**						
Dry ("hard")	0	0.0	-	-	-	-
Damp ("firm")	586	76.9	ref			
Wet ("squelchy")	176	23.1	0.80	0.23	2.78	0.722
Saturated ("boggy")	0	0.0	-	-	-	-
**Pasture quality (Last month of T2)**						
Lush (~ 90% leafy rye grasses)	399	52.4	ref			
Average (~ 50% rye grasses)	195	25.6	**1.76**	1.01	3.07	0.045
Poor (mostly stalk and weeds)	168	22.0	**4.08**	2.17	7.68	<0.001
**Pasture type (Last month of T2)**						
Permanent grassland	168	22.0	ref			
New grass ley	0	0.0	-	-	-	-
Mix permanent and new ley	594	78.0	**0.30**	0.15	0.60	0.001
**Sward height (Last month of T2)**						
Approx. 3 cm	418	54.9	ref			
Approx. 8 cm	344	45.1	1.99	0.84	4.73	0.120
Approx. > 8 cm	0	0.0	-	-	-	-
**Rainfall (calendar month of visit)**	762	100.0	1.00	0.97	1.02	0.725
**Temperature (calendar month of visit)**	762	100.0	1.12	0.05	25.94	0.944

CI: confidence interval for odds ratio; bold odds ratios are statistically significant at 0.05 as their CIs do not include 1; ref: baseline category for comparison.

Table S8. Univariable analyses of the associations with the development of WLD at foot-level for 815 observations of 286 ewes during transition period 3 (Jul 2020 – Sep 2020).

**Variable**	***n***	**%**	**Odds ratio**	**Lower 95% CI**	**Upper 95% CI**	***p***
Sheep-level variables						
**Age**						
< 4 years	505	62.0	ref			
≥ 4 years	310	38.0	**1.80**	1.09	2.98	0.022
**Change in BCS**						
Same condition	363	44.5	ref			
Gained condition	210	25.8	1.79	0.97	3.27	0.060
Lost condition	242	29.7	1.22	0.69	2.16	0.499
Foot-level variables						
**Foot position**						
Front	380	46.6	ref			
Back	435	53.4	**2.31**	1.60	3.35	<0.001
**Other feet develop WLD**						
None	204	25.0	ref			
One other	233	28.6	**4.06**	2.71	6.08	<0.001
Two others	237	29.1	**3.53**	2.37	5.26	<0.001
Three others	141	17.3	**11.07**	6.37	19.23	<0.001
**Clinical disease (First month of T3)**						
No FR disease present	807	99.0	ref			
ID and/or SFR present	8	1.0	6.67	0.44	100.45	0.170
**Clinical disease (Last month of T3)**						
No FR disease present	727	89.2	ref			
ID and/or SFR present	88	10.8	**2.69**	1.20	6.01	0.016
Farm-level variables						
**Flock size**						
< 500 ewes	208	25.5	ref			
≥ 500 ewes	607	74.5	0.85	0.47	1.54	0.597
**Vaccination status**						
Flock not vaccinated against footrot (Footvax®)	367	45.0	ref			
Flock vaccinated against footrot (Footvax®)	448	55.0	0.80	0.48	1.34	0.392
**Soil type**						
Loamy	208	25.5	ref			
Clay	402	49.3	0.73	0.39	1.36	0.322
Loamy/clay mix	205	25.2	1.15	0.56	2.35	0.703

**Pasture moisture (First month of T3)**						
Dry ("hard")	610	74.8	ref			
Damp ("firm")	205	25.2	1.37	0.78	2.41	0.267
Wet ("squelchy")	0	0.0	-	-	-	-
Saturated ("boggy")	0	0.0	-	-	-	-
**Pasture moisture (Last month of T3)**						
Dry ("hard")	0	0.0	-	-	-	-
Damp ("firm")	610	74.8	ref			
Wet ("squelchy")	205	25.2	1.37	0.78	2.41	0.267
Saturated ("boggy")	0	0.0	-	-	-	-
**Pasture quality (First month of T3)**						
Lush (~ 90% leafy rye grasses)	364	44.7	ref			
Average (~ 50% rye grasses)	208	25.5	1.05	0.73	1.50	0.791
Poor (mostly stalk and weeds)	243	29.8	**0.67**	0.48	0.94	0.019
**Pasture quality (Last month of T3)**						
Lush (~ 90% leafy rye grasses)	607	74.5	ref			
Average (~ 50% rye grasses)	208	25.5	1.29	0.74	2.26	0.372
Poor (mostly stalk and weeds)	0	0.0	-	-	-	-
**Pasture type (First month of T3)**						
Permanent grassland	243	29.8	ref			
New grass ley	0	0.0	-	-	-	-
Mix permanent and new ley	572	70.2	**1.51**	1.12	2.06	0.008
**Pasture type (Last month of T3)**						
Permanent grassland	243	29.8	ref			
New grass ley	0	0.0	-	-	-	-
Mix permanent and new ley	572	70.2	**1.90**	1.11	3.26	0.020
**Sward height (First month of T3)**						
Approx. 3 cm	367	45.0	ref			
Approx. 8 cm	448	55.0	0.85	0.59	1.21	0.359
Approx. > 8 cm	0	0.0	-	-	-	-
**Sward height (Last month of T3)**						
Approx. 3 cm	656	80.5	ref			
Approx. 8 cm	0	0.0	-	-	-	-
Approx. > 8 cm	159	19.5	1.16	0.62	2.16	0.646
**Rainfall (calendar month of visit)**	815	100.0	1.01	1.01	1.01	0.297
**Temperature (calendar month of visit)**	815	100.0	1.23	0.72	2.11	0.454

CI: confidence interval for odds ratio; bold odds ratios are statistically significant at 0.05 as their CIs do not include 1; ref: baseline category for comparison.

Table S9. Univariable analyses of the associations with the recovery from WLD at foot-level for 401 observations of 200 ewes during transition period 3 (Jul 2020 – Sep 2020).

**Variable**	***n***	**%**	**Odds ratio**	**Lower 95% CI**	**Upper 95% CI**	***p***
Sheep-level variables						
**Age**						
< 4 years	271	67.6	ref			
≥ 4 years	130	32.4	0.34	0.11	1.02	0.055
**Change in BCS**						
Same condition	177	44.1	ref			
Gained condition	130	32.4	0.46	0.15	1.36	0.161
Lost condition	94	23.4	1.09	0.35	3.41	0.877
Foot-level variables						
**Foot position**						
Front	228	56.9	ref			
Back	173	43.1	0.51	0.26	1.03	0.060
**Other feet recover from WLD**						
None	274	68.3	ref			
One other	82	20.4	**6.63**	3.84	11.46	<0.001
Two others	34	8.5	**6.81**	3.11	14.88	<0.001
Three others	11	2.7	**12.29**	3.09	48.85	<0.001
**Clinical disease (First month of T3)**						
No FR disease present	389	97.0	ref			
ID and/or SFR present	12	3.0	**1.21**	1.21	1.21	<0.001
**Clinical disease (Last month of T3)**						
No FR disease present	339	84.5	ref			
ID and/or SFR present	62	15.5	**0.16**	0.05	0.58	0.005
Farm-level variables						
**Flock size**						
< 500 ewes	96	23.9	ref			
≥ 500 ewes	305	49.4	0.48	0.16	1.42	0.185
**Vaccination status**						
Flock not vaccinated against footrot (Footvax®)	245	61.1	ref			
Flock vaccinated against footrot (Footvax®)	156	38.9	1.47	0.41	5.25	0.552
**Soil type**						
Loamy	96	23.9	ref			
Clay	198	49.4	0.60	0.19	1.91	0.391
Loamy/clay mix	107	26.7	0.32	0.09	1.18	0.088

**Pasture moisture (First month of T3)**						
Dry (“hard”)	294	73.3	ref			
Damp (“firm”)	107	26.7	0.48	0.17	1.35	0.162
Wet (“squelchy”)	0	0.0	-	-	-	-
Saturated (“boggy”)	0	0.0	-	-	-	-
**Pasture moisture (Last month of T3)**						
Dry (“hard”)	0	0.0	-	-	-	-
Damp (“firm”)	294	73.3	ref			
Wet (“squelchy”)	107	26.7	0.48	0.17	1.35	0.162
Saturated (“boggy”)	0	0.0	-	-	-	-
**Pasture quality (First month of T3)**						
Lush (~ 90% leafy rye grasses)	256	63.8	ref			
Average (~ 50% rye grasses)	96	23.9	**1.80**	1.09	2.97	0.021
Poor (mostly stalk and weeds)	49	12.2	**3.39**	1.81	6.36	<0.001
**Pasture quality (Last month of T3)**						
Lush (~ 90% leafy rye grasses)	305	76.1	ref			
Average (~ 50% rye grasses)	96	23.9	2.14	0.73	6.30	0.168
Poor (mostly stalk and weeds)	0	0.0	-	-	-	-
**Pasture type (First month of T3)**						
Permanent grassland	49	12.2	ref			
New grass ley	0	0.0	-	-	-	-
Mix permanent and new ley	352	87.8	**0.35**	0.18	0.70	0.003
**Pasture type (Last month of T3)**						
Permanent grassland	49	12.2	ref			
New grass ley	0	0.0	-	-	-	-
Mix permanent and new ley	352	87.8	**0.19**	0.05	0.72	0.015
**Sward height (First month of T3)**						
Approx. 3 cm	245	61.1	ref			
Approx. 8 cm	156	38.9	1.40	0.57	3.40	0.465
Approx. > 8 cm	0	0.0	-	-	-	-
**Sward height (Last month of T3)**						
Approx. 3 cm	252	62.8	ref			
Approx. 8 cm	0	0.0	-	-	-	-
Approx. > 8 cm	149	37.2	0.41	0.16	1.09	0.074
**Rainfall (calendar month of visit)**	401	100.0	**0.97**	0.97	0.98	<0.001
**Temperature (calendar month of visit)**	401	100.0	2.17	0.77	6.15	0.144

CI: confidence interval for odds ratio; bold odds ratios are statistically significant at 0.05 as their CIs do not include 1; ref: baseline category for comparison.
